# Laparoscopic enucleation: a safe and feasible treatment option for large (≥4 cm) benign or low-grade malignant pancreatic tumors

**DOI:** 10.3389/fmed.2025.1666758

**Published:** 2025-09-04

**Authors:** Chengqing Li, Yichen Yu, Wenyi Guo, Jiahao Wu, Jianwei Xu, Benzun Wei, Lei Wang

**Affiliations:** ^1^Department of Pancreatic Surgery, General Surgery, Qilu Hospital of Shandong University, Jinan, China; ^2^Pancreas Center, National Clinical Research Center for Cancer, State Key Laboratory of Druggability Evaluation and Systematic Translational Medicine, Tianjin Key Laboratory of Digestive Cancer, Tianjin’s Clinical Research Center for Cancer, Tianjin Medical University Cancer Institute and Hospital, Tianjin, China; ^3^Department of Hepatobiliary Surgery, Zibo Central Hospital, Zibo, China; ^4^Department of Pancreatic Surgery, General Surgery, Qilu Hospital of Shandong University, Jinan, China

**Keywords:** pancreatic tumor, laparoscopic enucleation, organ-sparing procedure, tumor size, pancreatic fistula, postoperative complications

## Abstract

**Background:**

The optimal surgical approach for large benign or low-grade malignant pancreatic tumors is controversial. The objective of this study was to evaluate the safety and feasibility of laparoscopic enucleation (LapEN) for large pancreatic tumors (≥4 cm).

**Methods:**

Patients who met the inclusion criteria at Qilu Hospital of Shandong University from January 2015 to May 2022 were retrospectively analyzed. First, the safety and feasibility of LapEN procedure were evaluated based on tumor diameter (≥4 cm or not). And then, we further compared the efficacy between LapEN and standard pancreatectomy [laparoscopic pancreaticoduodenectomy (LPD)/ laparoscopic distal pancreatectomy (LDP)] in patients with large tumors (≥4 cm).

**Results:**

Compared with patients with small tumors who underwent LapEN, there was no significant difference in rates of perioperative adverse events and postoperative complications in patients with large tumors who underwent LapEN, only postoperative hospital stays were prolonged. Among patients with large pancreatic tumors, comparison with standard pancreatectomy, LapEN achieved shorter operative time [(LapEN vs. LPD: 160.0 ± 41.4vs 396.8 ± 92.4 min, *p* < 0.001); (LapEN vs. LDP: 132.5 ± 53.0 vs. 223.1 ± 67.7 min, *p* < 0.001)] and less blood loss {[LapEN vs. LPD: 50 mL (range, 10–400 mL) vs. 300 mL (range, 50–1,000 mL), p < 0.001]; [LapEN vs. LDP: 40 mL (range, 5–300 mL) vs. 150 mL (range, 20–1,000 mL), *p* = 0.001]}. Particularly for large pancreatic head tumors, LapEN was superior to LPD in other terms of conversion rate, postoperative hospital stays, duration of fasting, pain score, and red blood cell transfusion rate.

**Conclusion:**

LapEN is a safe and feasible treatment option for large benign or low-grade malignant pancreatic tumors.

## Introduction

Consistently, complete surgical resection remains the mainstay of therapy for pancreatic tumors. Advances in imaging technology have increased the rate of detection for pancreatic tumors, and it implies that increasing numbers of patients will undergo pancreatic surgery. Conventional pancreatectomy such as pancreaticoduodenectomy and distal pancreatectomy were considered to be a highly invasive and complex type of abdominal procedure. Although these procedures can completely remove the tumor, excessive normal pancreatic parenchyma was unnecessarily removed and may result in pancreatic exocrine insufficiency and new-onset diabetes. Compared with standard pancreatectomy, pancreatic enucleation can reduce the removal of healthy pancreatic tissue and preserve exocrine and endocrine functions of the pancreas (1, 2). As a parenchyma-sparing procedure, pancreatic enucleation has been commonly performed as a safe procedure for pancreatic benign or low-grade malignant tumors such as pancreatic neuroendocrine tumor (PNET) (3), solid pseudopapillary tumor (SPT) (4), branch duct-intraductal papillary mucinous neoplasm (BD-IPMN) (5), serous cystic neoplasm (SCN) and mucinous cystic neoplasm (MCN) (6, 7).

With the development of laparoscopic instruments and techniques, laparoscopic enucleation (LapEN) has been widely accepted in many institutes (8). Compared with open procedures, LapEN could offer better short-term postoperative outcomes and postoperative pancreatic fistula (POPF) incidence rate (9–12). Currently, there is no clear indications of LapEN. For patients with benign or low-grade pancreatic tumors less than 4 cm, the safety and feasibility of the LapEN has been confirmed (3, 10, 13, 14). However, whether LapEN can also be performed safely in larger pancreatic tumors (≥4 cm) remains a controversial topic. In the face of large pancreatic tumors, LapEN were commonly selection with caution by surgeons. Therefore, this retrospective study was designed to explore the safety and feasibility of LapEN procedure to treat benign or low-grade malignant pancreatic tumors with a diameter ≥4 cm.

## Methods

### Patients collection and study design

**Figure 1:**
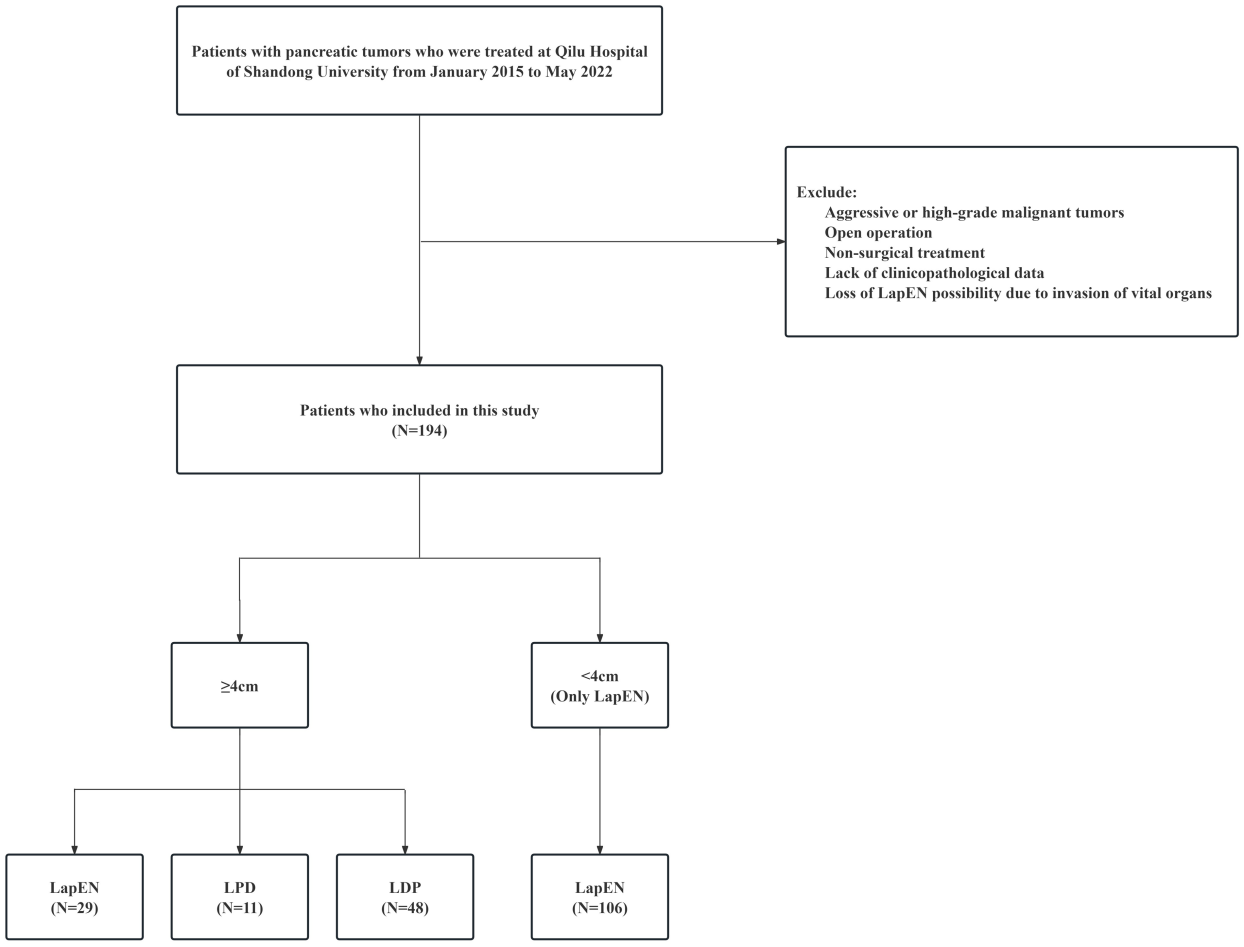
Flowchart of screening patients in this study. LapEN, laparoscopic enucleation; LPD, laparoscopic pancreaticoduodenectomy; LDP, laparoscopic distal pancreatectomy.

This study retrospectively analyzed patients with benign or low-grade malignant pancreatic tumors treated at Qilu Hospital from January 2015 to May 2022. The included patients must meet the following criteria: (a) diagnosed as benign or low-grade malignant tumors by pathology; (b) no major blood vessels, vital organs and common bile duct invasion (c) laparoscopic operation; (d) complete medical and follow-up data. Some patients were excluded if they were forced to undergo LPD and LDP due to vital organ invasion such as the splenic vessels and duodenum or a suspicion of high-grade malignancies based on intraoperative frozen pathology. The maximum tumor diameter was evaluated by preoperative imaging. Patients undergoing LapEN procedure were divided into large tumor group (≥4 cm) and small tumor group (<4 cm) according to tumor diameter. The screening process is shown in Figure 1. After screening, this retrospective study enrolled 194 patients, including 29 large tumor LapEN patients, 106 small tumor LapEN patients, 11 large tumor LPD patients and 48 large tumor LDP patients.

All data were from the medical records of Qilu Hospital of Shandong University. The serological parameters were collected within 3 days before the procedure. The imaging parameters were obtained based on a contrast enhanced computed tomography (CT) or magnetic resonance imaging (MRI) examination within 7 days prior to surgery. We evaluated the feasibility and safety of the LapEN procedure by intraoperative and postoperative parameters and complications. Intraoperative variables included American Society for of Anaesthesiologists (ASA) physical status classification, operative time, blood loss and pancreatic wound suture, obtained from the operative notes written by the surgeon. The Postoperative hospital stays, duration of fasting, visual analogue scale (VAS), red blood cell transfusion rate, occurrence of clinically relevant postoperative pancreatic fistula (CR-POPF), post-pancreatectomy hemorrhage (PPH) and delayed gastric emptying (DGE) were assessed to verify the safety of the operation. In addition, data on new-onset diabetes, exocrine insufficiency, and tumor recurrence were obtained through follow-up. Postoperative follow-up data were conducted by telephone. All patients were followed up for a minimum of 6 months.

This study was conducted according to the principles of the Declaration of Helsinki. All patients signed written informed consent. The analysis of patient data was approved by the Ethics Committee of Qilu Hospital of Shandong University.

### Surgical procedure

**Figure 2:**
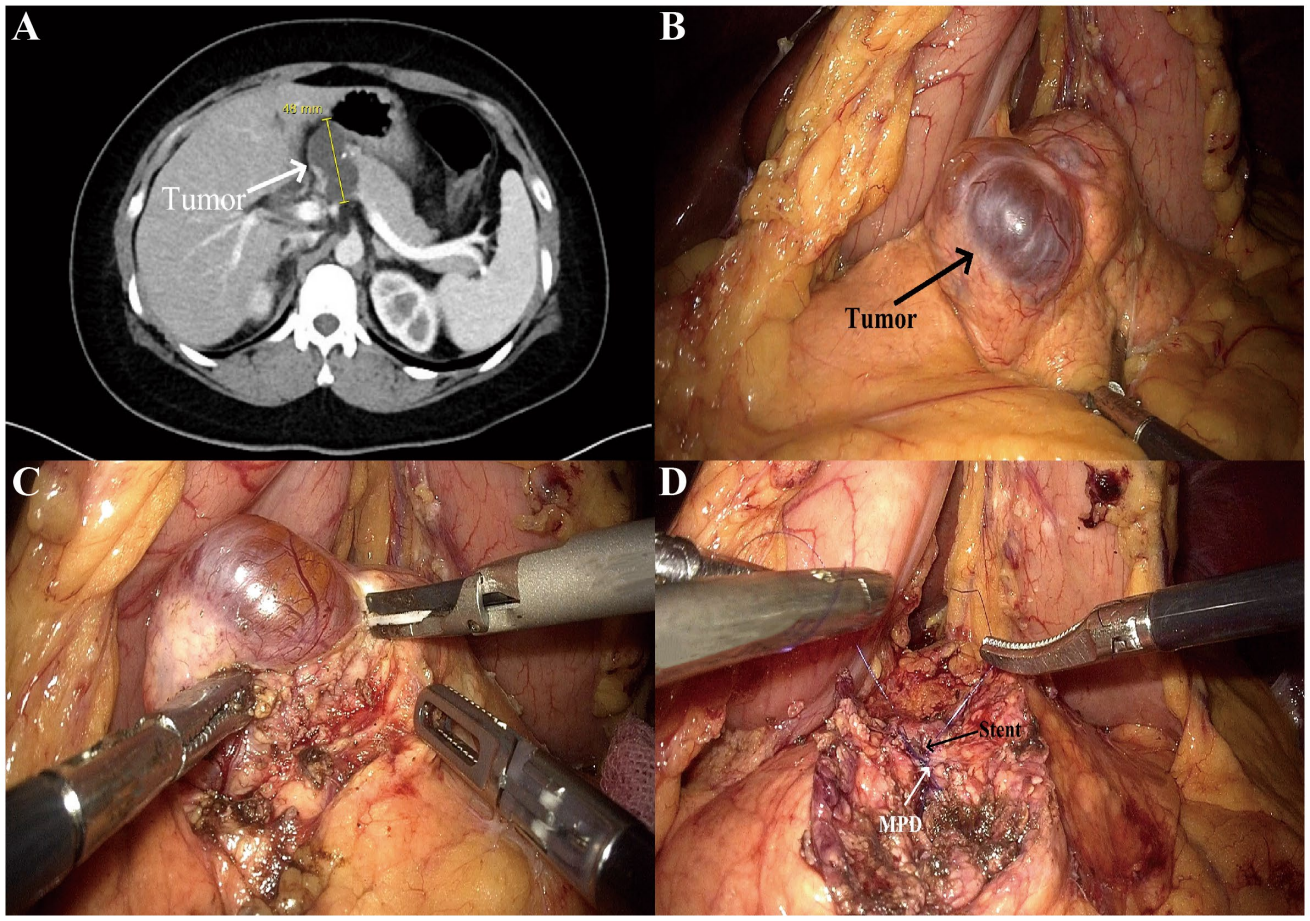
The procedure of laparoscopic enucleation for a case of large pancreatic tumor. **(A)** CT imaging revealed a large cystic tumor (4.8 cm in maximal diameter) located in the pancreatic neck. **(B)** A large cystic pancreatic tumor was identified by laparoscopy. **(C)** The tumor was enucleated from the pancreatic parenchyma. **(D)** The MPD injury was repaired with stent placement. MPD, main pancreatic duct.

LapEN was performed as we have previously reported (15, 16). The surgical procedure for LapEN of large tumors was shown in Figure 2. The patient was placed in the supine position on the operating table with the legs spread apart. The main surgeon was on the right side of the patient and the assistant was on the left side of the patient. The laparoscopic assistant stood between the patient’s legs and was responsible for adjustment of the laparoscope. A total of 5 trocars were used during the procedure. A 10-mm trocar was inserted lower edge of the umbilical region and used for observing. A 12-mm trocar located in the right mid-clavicular line, and a 5-mm trocar located at the right anterior axillary line. Symmetrically, another two 5-mm trocars located in the left mid-clavicular line and left anterior axillary line. The whole abdominal cavity was explored first. Next, the gastrocolic ligament was opened to reveal the anterior aspect of the pancreas. The lesion was enucleated from the pancreatic parenchyma (Figure 2C). Surgeons should pay attention to protect the main pancreatic duct (MPD) and major blood vessels during the operation. If an injury of the MPD happened during the resection, we used PDS II (polydioxanone) synthetic absorbable suture for repair, and insert a stent if necessary (Figure 2D). For tumors whose margins could not be determined, we usually used intraoperative ultrasound for evaluation. Finally, the pancreatic wound was carefully examined and one or two drainage tubes were placed there. Tumor specimens were sent to the pathology department for examination.

LPD and LDP were performed under the standardized surgical procedure. If the spleen would be preserved in LDP, we would perform the Warshaw (17) or Kimura (18) procedure.

### Definition and classification of complications

POPF was defined based on the 2016 International Study Group of Pancreatic Fistula (ISGPF) definition and classification (19). POPF was defined as an amylase level in any measurable excreted fluid that was 3 times higher than the upper limit of the institution’s normal serum amylase level. The upper limit of serum amylase in our hospital is 105 U/L. The original “grade A” POPF, the biochemical fistula, was no longer considered an actual postoperative complication. Grade B and C POPF are classified as CR-POPF.

According to the ISGPF, DGE was defined as reinsertion of a nasogastric tube 3 days after surgery or failure to start to oral diet 7 days after surgery (20). The definition of PPH was based on ISGPF, Included grade B (intervention is required) and C (patient in critical condition) (21). The diagnosis of new-onset diabetes after surgery was based on the 2021 American Diabetes Association (ADA) Standards of Medical Care in Diabetes (22). Exocrine insufficiency was defined as pancreatic enzyme insufficiency requiring pancreatic enzyme supplementation therapy.

### Statistical methods

Statistical analyses were performed by SPSS 26 (SPSS, Inc., Chicago, IL). For measurement data, the Kolmogorov–Smirnov and Shapiro–Wilk normality tests were used to evaluate its normality. Normally distributed values were described by the mean ± standard deviation, and nonnormally distributed values were described by the median and the range. Student’s *t*-test and Mann–Whitney U-test was performed to compare measurement data. Categorical data were recorded as percentages, and were compared by Pearson Chi square or Fisher’s exact tests. All *p* values were two-sided, and *p* < 0.05 was considered statistically significant.

## Results

### Clinicopathologic characteristics of the enrolled patients

A total of 194 patients was enrolled in this study. The baseline characteristics of all enrolled patients are listed in Table 1. Among them, 135 patients underwent LapEN, 11 patients underwent LPD and 48 patients underwent LDP. In the overall, the median age of patients was 44 (11–79) years, and 147 (75.8%) patients were females. The median tumor size was 3.0 cm (range, 0.6–12.0 cm), and 87 (44.8%) patients had tumors located in the head of the pancreas. All tumors were surgically removed completely, and a postoperative pathological examination was performed to diagnose. The pathological diagnosis of all tumors was shown in Table 2. SCN (25%), SPT (30.7%), and MCN (20.5%) account for the majority of patients with large pancreatic tumors. Unlike the former, the proportion of PNET (43.4%) was highest in the small tumor group. At the end of follow-up, all patients were alive.

**Table 1:** The baseline characteristics of all enrolled patients.

Variables	Enrolled patients *n* = 194
Age (years), Median (range)	44 (11–79)
Sex (Male/ Female)	47/147
BMI (≥24 kg/m^2^/<24 kg/m^2^)	97/97
Smoking habit (Yes/ No)	19/175
Drinking habit (Yes/ No)	20/174
Hypertension (Yes/ No)	35/159
Diabetes (Yes/ No)	16/178
Abdominal pain (Yes/ No)	46/148
ASA score, Median (range)	2 (1–3)
Tumor size (cm), Median (range)	3 (0.6–12)
Location (Head/ Body and tail)	87/107
Cystic tumors (Yes/ No)	117/77
Hemoglobin(g/L), Mean± SD	130.3 ± 17.0
Platelet(10^9^/L), Mean± SD	245.9 ± 61.9
Neutrophil (10^9^/L), Mean± SD	3.33 ± 1.25
Lymphocyte(10^9^/L), Mean± SD	1.72 ± 0.53
Albumin(g/L), Mean± SD	44.0 ± 5.4
Total bilirubin(μmol/L), Mean± SD	10.5 ± 5.3
Pathology (PNET / Other)	52/142
Surgical procedure (LapEN/ Standard pancreatectomy)	135/59

BMI, body mass index; ASA, American society for of anaesthesiologists; PNET, pancreatic neuroendocrine tumor; LapEN, laparoscopic enucleation.

**Table 2:** Pathological diagnosis of 194 patients in this study.

Pathological diagnosis	Large tumor(≥4 cm)			Small tumor(<4 cm)
	LapEN	LPD	LDP	LapEN
PNET	1	3	2	46
SCN	7	-	15	22
SPT	10	2	15	12
Pseudocyst	4	-	3	8
MCN	5	-	13	5
Inflammatory mass	1	2	-	4
Lymphangioma	-	-	-	4
BD-IPMN	-	4	-	2
Castleman	1	-	-	1
Hamartoma	-	-	-	2
Total	29	11	48	106

LapEN, laparoscopic enucleation; LPD, laparoscopic pancreaticoduodenectomy; LDP, laparoscopic distal pancreatectomy; PNET, pancreatic neuroendocrine tumor; SCN, serous cystic neoplasm; SPT, solid pseudopapillary tumor; MCN, mucinous cystic neoplasm; BD-IPMN, branch duct-intraductal papillary mucinous neoplasm.

### Safety assessment of LapEN based on tumor diameter

A total of 135 patients underwent LapEN procedure, 29 (21.5%) were classified in the large tumor group (≥4 cm) and 106 (78.5%) to the small tumor group (<4 cm) based on tumor diameter. The clinicopathologic characteristics of patients undergoing LapEN procedure were listed in Table 3. The mean tumor diameter was 5.7 ± 1.7 cm in the large tumor group, and was 2.0 ± 0.7 cm in the small tumor group. One case of MCN had the largest tumor with a diameter of 10 cm. Patients in the large tumor group had a significantly greater proportion of cystic lesions than in the small tumor group (79.3% vs. 48.1%, *p* = 0.003). Postoperative pathology showed that the proportion of PNET was significantly lower in patients with large tumors than in those with small tumors (3.4% vs. 43.4%, *p* < 0.001). The mean operating time for LapEN procedure was 144.9 ± 59.9 min. Because of the difficulty in intraoperative search for tumors, 3 (2.2%) patients were converted to open surgery. There was no difference between both groups in terms of operative time (150.5 ± 46.7 vs. 143.3 ± 63.2 min, *p* = 0.569), intraoperative blood loss [50 mL (5–400 mL) vs. 50 mL (2–300 mL), *p* = 0.545], and conversion rate (0% vs. 2.8%, *p* = 1.000). In the perioperative setting, large tumor only seemed to lengthen hospital stay after the operation [8 days (3–21 days) vs. 6 days (2–30 days), *p* = 0.009]. The incidence of long-term and short-term complications for patients in two subgroups was not significantly different. CR-POPF was the most common postoperative complication. The CR-POPF rate in LapEN cohort was 21.5%, including 28 cases were grade B and one case was grade C. Only one patient was found to have a tumor recurrence at 6 months postoperatively and was prepared to undergo surgery at a later date.

**Table 3:** Comparison of patients undergoing LapEN according to tumor size (≥4 cm or not).

Variables	Large tumor (≥4 cm) *n* = 29	Small tumor (<4 cm) *n* = 106	*p* value
Tumor size (cm), Mean± SD	5.7 ± 1.7	2.0 ± 0.7	<0.001
Age (years), Median (range)	36 (12–63)	51 (11–79)	0.001
Sex (Male/ Female)	5/24	32/74	0.166
BMI (≥24 kg/m^2^/<24 kg/m^2^)	13/16	59/47	0.300
Smoking habit (Yes/ No)	1/28	13/93	0.168
Drinking habit (Yes/ No)	2/27	13/93	0.415
Hypertension (Yes/ No)	4/25	21/85	0.460
Diabetes (Yes/ No)	1/28	8/98	0.433
Abdominal pain (Yes/ No)	9/20	24/82	0.351
ASA score, Median (range)	2 (1–2)	2 (1–3)	0.140
Location (Head/ Body and tail)	19/10	57/49	0.259
Cystic tumors (Yes/ No)	23/6	51/55	0.003
Hemoglobin(g/L), Mean± SD	128.7 ± 13.3	132.0 ± 18.4	0.369
Platelet(10^9^/L), Mean± SD	265.0 ± 66.5	244.6 ± 62.9	0.129
Neutrophil (10^9^/L), Mean± SD	3.45 ± 1.55	3.34 ± 1.10	0.655
Lymphocyte(10^9^/L), Mean± SD	1.79 ± 0.58	1.73 ± 0.52	0.575
Albumin(g/L), Mean± SD	44.5 ± 3.1	44.4 ± 4.9	0.968
Total bilirubin(μmol/L), Mean± SD	10.4 ± 4.3	10.7 ± 5.2	0.732
Pathology (PNET / Other)	1/28	46/60	<0.001
Operative time (min), Mean± SD	150.5 ± 46.7	143.3 ± 63.2	0.569
Blood loss (ml), Median (range)	50 (5–400)	50 (2–300)	0.545
Conversion (Yes/ No)	0/29	3/103	1.000
Postoperative hospital stays (days), Median (range)	8 (3–21)	6 (2–30)	0.009
Duration of fasting(days), Median (range)	3 (1–11)	3 (1–25)	0.312
VAS pain score, Median (range)	3 (1–4)	3 (1–6)	0.398
Transfusion of red cells (Yes/ No)	1/28	4/102	1.000
CR-POPF (Yes/ No)	8/21	21/85	0.366
Post-pancreatectomy hemorrhage (Yes/ No)	1/28	2/104	0.519
Delayed gastric emptying (Yes/ No)	1/28	3/103	1.000
Clavien-Dindo grade (≥III/<III)	2/27	8/98	0.906
New-onset diabetes (Yes/ No)	1/28	0/106	0.215
Exocrine insufficiency (Yes/ No)	1/28	1/105	0.385
Recurrence (Yes/ No)	1/28	0/106	0.215

LapEN, laparoscopic enucleation; BMI, body mass index; ASA, American society for of anaesthesiologists; PNET, pancreatic neuroendocrine tumor; VAS, visual analogue scale; CR-POPF, clinically relevant postoperative pancreatic fistula.

### Comparative analysis of patients undergoing LapEN and LPD/LDP procedures

The standard procedure for pancreatic head tumors is the pancreaticoduodenectomy, and distal pancreatectomy is the standard procedure for pancreatic body and tail tumors. For those patients with large tumors (≥4 cm) who did not undergo LapEN surgery, other appropriate procedures were selected according to the location of the tumor, including 11 cases of LPD and 48 cases of LDP. To compare the safety and feasibility of different procedures for patients with large pancreatic tumors, we classified patients according to tumor location and then analyzed separately. The clinicopathologic characteristics of these patients were shown in Tables 4, 5.

**Table 4:** Comparative analysis of patients undergoing LapEN and LPD procedures.

Variables	Large pancreatic head tumors (≥4 cm)		*p* value
	LapEN group *n* = 19	LPD group *n* = 11	
Age (years), Median (range)	30 (12–57)	44 (23–63)	0.053
Sex (Male/ Female)	4/15	5/6	0.160
BMI (≥24 kg/m^2^/<24 kg/m^2^)	7/12	4/7	0.979
Smoking habit (Yes/ No)	1/18	3/8	0.126
Drinking habit (Yes/ No)	1/18	2/9	0.537
Hypertension (Yes/ No)	1/18	1/10	1.000
Diabetes (Yes/ No)	0/19	2/9	0.126
Abdominal pain (Yes/ No)	5/14	4/7	0.563
ASA score, Median (range)	2 (1–2)	2 (1–3)	0.160
Cystic tumors (Yes/ No)	14/5	5/6	0.122
Hemoglobin(g/L), Mean± SD	128.9 ± 11.5	131.6 ± 18.2	0.634
Platelet(10^9^/L), Mean± SD	287.5 ± 60.1	256.0 ± 48.7	0.150
Neutrophil granulocyte(10^9^/L), Mean± SD	3.77 ± 1.71	4.04 ± 2.01	0.698
Lymphocyte(10^9^/L), Mean± SD	1.91 ± 0.63	1.67 ± 0.29	0.252
Albumin(g/L), Mean± SD	44.5 ± 2.6	41.7 ± 8.4	0.303
Total bilirubin(μmol/L), Mean± SD	10.3 ± 3.9	13.49 ± 10.11	0.335
Pathology (PNET / Other)	0/19	3/8	0.041
Operative time(min), Mean± SD	160.0 ± 41.4	396.8 ± 92.4	<0.001
Blood loss (ml), Median (range)	50 (10–400)	300 (50–1,000)	<0.001
Conversion (Yes/ No)	0/19	4/7	0.012
Postoperative hospitalization stays (days), Median (range)	9 (3–21)	14 (10–26)	0.006
Duration of fasting(days), Median (range)	3 (1–11)	6 (4–13)	<0.001
VAS pain score, Median (range)	3 (2–4)	4 (3–6)	0.001
Transfusion of red blood cells (Yes/ No)	0/19	6/5	0.001
CR-POPF (Yes/ No)	5/14	4/7	0.563
Post-pancreatectomy hemorrhage (Yes/ No)	0/19	1/10	0.367
Delayed gastric emptying (Yes/ No)	1/18	2/9	0.537
Clavien-Dindo grade (≥III/<III)	0/19	1/10	0.367
New-onset diabetes (Yes/ No)	0/19	1/10	0.367
Exocrine insufficiency (Yes/ No)	0/19	4/7	0.012
Recurrence (Yes/ No)	0/19	0/11	1.000

LapEN, laparoscopic enucleation; LPD, laparoscopic pancreaticoduodenectomy; BMI, body mass index; ASA, American society for of anaesthesiologists; PNET, pancreatic neuroendocrine tumor; VAS, visual analogue scale; CR-POPF, clinically relevant postoperative pancreatic fistula.

**Table 5:** Comparative analysis of patients undergoing LapEN and LDP procedures.

**Variables**	Large pancreatic body and tail tumors (≥4 cm)		*p* value
	LapEN group *n* = 10	LDP group *n* = 48	
Age (years), Median (range)	44 (25–63)	39 (20–68)	0.387
Sex (Male/ Female)	1/9	5/43	0.969
BMI (≥24 kg/m^2^/<24 kg/m^2^)	6/4	21/27	0.349
Smoking habit (Yes/ No)	0/10	2/46	1.000
Drinking habit (Yes/ No)	1/9	3/45	0.541
Hypertension (Yes/ No)	3/7	9/39	0.424
Diabetes (Yes/ No)	1/9	5/43	1.000
Abdominal pain (Yes/ No)	4/6	9/39	0.143
ASA score, Median (range)	2 (1–2)	2 (1–2)	0.255
Cystic tumors (Yes/ No)	9/1	38/10	0.427
Hemoglobin(g/L), Mean± SD	128.3 ± 16.9	127.2 ± 15.5	0.634
Platelet(10^9^/L), Mean± SD	222.3 ± 58.5	236.8 ± 58.1	0.477
Neutrophil granulocyte(10^9^/L), Mean± SD	2.86 ± 1.02	3.08 ± 1.13	0.571
Lymphocyte(10^9^/L), Mean± SD	1.57 ± 0.40	1.67 ± 0.56	0.570
Albumin(g/L), Mean± SD	44.3 ± 3.9	43.4 ± 6.7	0.710
Total bilirubin(μmol/L), Mean± SD	10.5 ± 5.2	9.5 ± 4.4	0.537
Pathology (PNET / Other)	1/9	2/46	0.439
Operative time(min), Mean± SD	132.5 ± 53.0	223.1 ± 67.7	<0.001
Blood loss (ml), Median (range)	40 (5–300)	150 (20–1,000)	0.001
Conversion (Yes/ No)	1/9	3/45	0.541
Postoperative hospitalization stays (days), Median (range)	7 (3–17)	8 (4–16)	0.716
Duration of fasting (days), Median (range)	3 (1–6)	4 (2–8)	0.199
VAS pain score, Median (range)	3 (2–4)	4 (2–6)	0.065
Transfusion of red blood cells (Yes/ No)	1/9	10/38	0.427
CR-POPF (Yes/ No)	3/7	13/35	0.851
Post-pancreatectomy hemorrhage (Yes/ No)	1/9	1/47	0.318
Delayed gastric emptying (Yes/ No)	0/10	2/46	1.000
Clavien-Dindo grade (≥III/<III)	2/19	2/46	0.134
New-onset diabetes (Yes/ No)	1/19	13/35	0.251
Exocrine insufficiency (Yes/ No)	1/19	5/43	1.000
Recurrence (Yes/ No)	1/19	0/48	0.172

LapEN, laparoscopic enucleation; LDP, laparoscopic distal pancreatectomy; BMI, body mass index; ASA, American society for of anaesthesiologists; PNET, pancreatic neuroendocrine tumor; VAS, visual analogue scale; CR-POPF, clinically relevant postoperative pancreatic fistula.

In large pancreatic head tumors cohort, 19 patients underwent LapEN and 11 patients underwent LPD. There were no significant differences in the demographic parameters between the both subgroups (Table 4). Compared to the LapEN group, the operative time was significantly longer (396.8 ± 92.4 vs. 160.0 ± 41.4 min, *p* < 0.001) and intraoperative blood loss increased significantly [300 mL (range, 50–1,000 mL) vs. 50 mL (range, 10–400 mL), *p* < 0.001] in LPD group. In addition, the conversion to open surgery rate of LPD was significantly higher than that of LapEN (45.5% vs. 0%, *p* = 0.012). In terms of postoperative parameters, postoperative hospital stays [9 days (range, 3–21 days) vs. 14 days (range, 10–26 days), *p* = 0.006] and duration of fasting [3 days (range, 1–11 days) vs. 6 days (range, 4–13 days), *p* < 0.001] for patients undergoing LapEN were significantly shorter than for patients undergoing LPD procedure. The red blood cell transfusion rate (0% vs. 54.5%, *p* = 0.001) and postoperative pain scores [3 (range, 2–4) vs. 4 (range, 3–6), *p* = 0.001] were significantly lower in LapEN than in LPD. There were no significant differences in the incidence of CR-POPF and other short-term complications between both subgroups. In terms of long-term complications, the incidence of exocrine insufficiency was significantly higher in patients undergoing LPD (36.4% vs. 0%, *p* = 0.012).

In large pancreatic body and tail tumors cohort, 10 patients underwent LapEN and 48 patients underwent LDP. The subgroups did not significantly differ on the demographic parameters (Table 5). Of the patients who underwent LDP, 12 cases underwent intraoperative splenectomy, 20 cases underwent Kimura procedure and 16 cases underwent Warshaw procedure. During the LDP, the operative time was significantly prolonged (223.1 ± 67.7 vs. 132.5 ± 53.0 min, *p* < 0.001) and intraoperative blood loss increased significantly [150 mL (range, 20–1,000 mL) vs. 40 mL (range, 5–300 mL), *p* = 0.001] than LapEN. In terms of short-term and long-term complications, there were no significant differences between both subgroups.

## Discussion

LapEN can avoid unnecessary resection of normal pancreatic tissue, and preserve exocrine and endocrine functions of the pancreas. Different from the standard pancreatectomy (LPD/LDP), LapEN does not involve the removal of the main pancreatic duct and common bile duct, avoiding complex reconstruction (23). For a long time, the indications of LapEN are not well-defined. Conventional wisdom suggests that LapEN can be performed safely for pancreatic tumors around 4 cm in diameter, but LapEN should be considered cautiously for larger pancreatic tumors (24, 25). However, when diagnosis was confirmed, tumor grown probably larger than 4 cm and even larger (26). In addition, a European guideline recently concluded that both a MCN and IPMN <40 mm can be treated conservatively if other risk factors are absent (27). Therefore, the indications for LapEN should be expanded as appropriate. At present, there are only a few sporadic reports available on LapEN being performed for large pancreatic tumors, and lacked control groups (28, 29). To evaluate the safety and feasibility of LapEN for large pancreatic tumors (≥4 cm), we designed this study.

In this retrospective study, we analyzed the outcomes of LapEN for benign or low-grade malignant pancreatic tumors of different diameters. A total of 135 patients underwent LapEN, the incidence of CR-POPF was 21.5% (29/135), which is an acceptable result. In addition, there were 3 (2.2%) patients had PPH and 4 patients had DGE (3.0%) after LapEN. During the follow-up period, one patient had new-onset diabetes, one patient had exocrine insufficiency, one patient appeared tumor recurrence and no patients have died. Overall, postoperative outcomes were excellent. Compared with patients with small tumors undergoing LapEN, those with large tumors only experienced prolonged hospital stays after LapEN. However, other perioperative adverse events and complication rates were not significantly different between them. Next, we further analyzed whether LapEN was superior to standard pancreatectomy (LPD/LDP) for patients with large benign or low-grade malignant pancreatic tumors. After comprehensive comparison, compared to standard pancreatectomy, LapEN may offer better perioperative outcomes and has certain strengths in terms of short and long-term complication rate. These results showed that LapEN procedure can be performed as a safe and feasible treatment option for large benign or low-grade malignant pancreatic tumors.

Normally, LapEN is mainly suitable for benign or low-grade malignant pancreatic tumors such as PNET (3), SPT (4), BD-IPMN (5), SCN and MCN (6, 7). Some studies have reported that LapEN can also be performed to treat isolated pancreatic metastases from renal cell carcinoma, but the recurrence rate after limited resection is higher than that of radical resection (30). Notably, when tumor size increases, certain tumors like MCN, BD-IPMN and PNET have more potential to undergo malignant transformation and be involved in lymph node metastasis (27, 31, 32). If there is any suspicion of aggressive malignancy, the LapEN will no longer apply. Preoperative imaging assessment and routine intraoperative frozen sectioning are indispensable to ensure the successful completion of the surgery (4). Patients who have underwent LapEN should require regular postoperative review to monitor recurrence. Dalla Valle et al. analyzed 1,223 cases and found that the mean recurrence rate after enucleation was 2.2% (10). In our study, only 1 (0.7%) case of MCN was identified as a recurrence at postoperative follow-up. Altogether, the recurrence rate after enucleation is acceptable.

POPF is the most important post-operative complication of LapEN. In the past, LapEN was considered to have a higher risk of POPF than open procedure (12, 33). However, recent meta-analyses indicated that LapEN did not increase the risk of POPF. On the contrary, provide the patients with better short-term outcomes, including shorter operative time, smaller incisions and shorter hospital stay (10, 12). The key point to prevent POPF during the operation is to avoid injuring the MPD (23). Crippa et al. suggested that the lesion must be at least 2–3 mm from the MPD to ensure the safety of the LapEN procedure (34). However, Strobel et al. reported that tumors can be safely enucleated even if they were close to the MPD (35), our previous study also supported this viewpoint (15). Intraoperative ultrasound is an important tool to assist in determining the anatomical relationship between tumor with the main pancreatic duct, and it can guide surgeons in choosing the appropriate surgical technique (36).

Several previous studies have found that enucleation provided shorter operative time, less blood loss and shorter hospital stay as compared to standard pancreatectomy. Particularly in terms of long-term complications, enucleation showed a lower incidence of new-onset diabetes and pancreatic exocrine insufficiency (2, 37). Although our study also showed similar results, only LapEN showed a statistically significant difference in exocrine insufficiency rate exocrine insufficiency rate LPD. Part of this inconsistency may be due to small sample sizes in this study.

This study has several limitations. First, as a single-center study with a relatively small sample size, some baseline heterogeneity existed between groups. Second, the follow-up duration was limited in our cohort, resulting in a lack of long-term outcome data. These limitations suggest that the interpretation of results might be made with caution. Therefore, large-scale, multicenter prospective studies with extended follow-up are warranted to further validate the safety and efficacy of LapEN for large benign or low-grade malignant pancreatic neoplasms.

In conclusion, LapEN a safe and feasible technique for benign or low-grade malignant pancreatic tumors larger than 4 cm. Compared with standard pancreatectomy (LPD/LDP), LapEN presents evident perioperative advantages such as shorter operative time and less blood loss. Notably, strict adherence to surgical indications of LapEN must be required, and the procedure should be performed by experienced pancreatic surgical teams. In the future, more large sample and multi-center studies are needed to further verify its safety and feasibility.

## Data Availability

The original contributions presented in the study are included in the article/supplementary material, further inquiries can be directed to the corresponding authors.
